# Clinical implications of a history of pre‐eclampsia in women with type two diabetes mellitus

**DOI:** 10.1111/dme.70046

**Published:** 2025-04-16

**Authors:** Madeline C. Pearson, Huan Wang, Colin E. Murdoch, Alexander S. F. Doney

**Affiliations:** ^1^ School of Medicine University of Dundee Dundee UK

**Keywords:** cardiovascular disease, chronic kidney disease, diabetic retinopathy, microvascular complications, pre‐eclampsia, type 2 diabetes, women's health

## Abstract

**Aims:**

The consequences of pre‐eclampsia (PE) are not limited to pregnancy; a single episode predisposes mothers to serious future health outcomes. Little is known about how PE impacts the course of type 2 diabetes (T2D) and associated microvascular diseases.

**Methods:**

The Scottish Care Information for Diabetes data for individuals with diabetes in NHS Tayside and Fife was linked with Scottish maternity morbidity records. A nested case–control study compared BMI, HDL cholesterol, systolic blood pressure(SBP) and HbA1c measures at T2D diagnosis between women with prior pregnancies from 1921 to 2022 affected or unaffected by PE using linear regression and adjusted for the other aforementioned variables. Cox regression models assessed how a PE history influenced the risk of future microvascular complications following T2D diagnosis.

**Results:**

In total, 6055 women were eligible: 726 (12%) had a PE‐pregnancy and 5329 (88%) had only PE‐free pregnancies, with ~20 years between pregnancy and T2D diagnosis. At T2D diagnosis, women with a PE history had higher BMI (38.3 kg/m^2^ vs. 35.7 kg/m^2^, *p* < 0.001), higher SBP (139 mmHg vs. 135 mmHg, *p* < 0.001), lower HDL cholesterol (1.18 mmol/L vs. 1.21 mmol/L, *p* = 0.002) and were diagnosed 3.2 years earlier (*p* < 0.001). A PE history was associated with increased microvascular disease risk (HR 1.26 95% CI 1.12–1.42, *p* < 0.001): diabetic retinopathy (HR 1.22 95% CI 1.07–1.38, *p* = 0.003); chronic kidney disease (HR 1.35 95% CI 1.06–1.71, *p* = 0.016); diabetic proteinuric kidney disease (HR 2.45 95% CI 1.03–5.81, *p* < 0.001). Women with a PE history were ~5 years younger when they developed cardiovascular disease (55.7 years vs. 60.6 years, *p* < 0.001) and all‐cause death (60.1 years vs. 65.6 years, *p* < 0.0001).

**Conclusions:**

At T2D diagnosis, women with a PE history are younger, with more severe clinical presentations and increased risk of developing T2D microvascular complications. This highlights the crucial need for changes to the long‐term care of this high‐risk group, with aggressive risk‐factor management and continued clinical assessment.

**What's new?**

**What is already known about the subject?**

- Pre‐eclampsia (PE) is known to increase the risk of type 2 diabetes (T2D) development, but little is known about how a history of it impacts the clinical presentation and course of T2D.

**What has this study found?**

- At point of diagnosis with T2D women with a history of PE in pregnancy are younger, and have a more severe clinical presentation, with a higher BMI and blood pressure. Subsequently they are at greater risk of developing microvascular complications—retinopathy, chronic kidney disease and proteinuric kidney disease.

**What are the implications of the study?**

- A history of PE should be elucidated at first presentation of women with T2D, as they form a high‐risk sub‐group that may benefit from closer monitoring, early microvascular assessment and rapid intervention.

## 1 INTRODUCTION

Pre‐eclampsia (PE) in pregnancy is a major cause of maternal and fetal morbidity and mortality worldwide. While PE usually resolves with placental delivery, there is growing evidence that a PE history is associated with several serious health conditions that may only develop decades later. For example, PE is recognised to be a risk factor for adverse cardiovascular outcomes, carrying a fourfold increased risk of heart failure and doubling the risk of future strokes, coronary artery disease and a cardiovascular cause of death.^1^ A PE history has also been linked to greater lifetime risks for venous thromboembolism,^2^ end‐stage renal disease^3^ and cognitive decline, particularly affecting executive functioning, in later life.^4^

A PE‐history is an independent risk factor for the development of type 2 diabetes, and pre‐existing diabetes predisposes the development of PE.^5^ Diabetes is associated with long‐term micro and macrovascular complications, including retinopathy, nephropathy and peripheral vascular disease. In patients with pre‐existing diabetes, an episode of PE increases the risk of these complications.^6^ However, the majority of women diagnosed with PE during pregnancy do not have a prior diagnosis of diabetes, and little is known about how a PE‐history impacts the onset and clinical course of type 2 diabetes.

The UK Government's Women's Health Plans^7^, ^8^ have called for improved understanding of the differences in how men and women are affected by diabetes, with acknowledgement of the specific risks surrounding pregnancy and the growing recognition that health during pregnancy may impact health outcomes even many decades later. We therefore investigated a cohort of women diagnosed with type 2 diabetes following pregnancy and compared the clinical features at the time of diabetes diagnosis and risk of developing microvascular complications in women with pregnancies complicated by PE and those with PE‐free pregnancies. Our hypothesis was that a history of PE during pregnancy would increase the risk of developing microvascular complications of type 2 diabetes.

## 2 METHODS

### 2.1 Study population

The source population for this study comprised all individuals with diabetes, both currently and historically (July 1977–March 2022) managed in NHS Scotland Tayside and Fife health boards. Healthcare data for this population were obtained from the Scottish Care Information for Diabetes (SCI‐Diabetes) data and linked anonymously with national Scottish Morbidity Records for maternity inpatient and day cases (SMR02) made available by Public Health Scotland (PHS). All data were provided anonymised by the Health Informatics Centre at the University of Dundee under standard research governance operating procedures^9^ in a Trusted Research Environment with the Caldicott Guardian approval. Anonymised patient identifiers were used to link data from other PHS and Health Informatics Centre‐provided datasets (Table S1) to determine both the clinical status at the time of type‐2 diabetes diagnosis and the subsequent development of diabetic complications.

The study population was defined as all women in NHS Tayside and Fife who received a diagnosis of type 2 diabetes—using the SCI‐Diabetes dataset—and had a pregnancy recorded in SMR02 data from 1974 to 2023. Women with pre‐existing type 2 diabetes at the time of any of their pregnancies were excluded. ICD‐10 and ICD‐9 codes were used to label cases as women with a history of PE in pregnancy—pre‐eclampsia (ICD10 O11, O14 and ICD9 6424, 6425, 6427) and eclampsia (ICD10 O15 and ICD9 6426) (Table S2). All other women, with no history of PE during any pregnancy, were controls, forming a nested case–control study cohort.

### 2.2 Clinical status at diabetes diagnosis

To determine the clinical status of patients at the point of type 2 diabetes diagnosis, a mean of BMI, blood pressure, HDL cholesterol, total cholesterol and HbA1c measurements taken within 2 years either side of the type 2 diabetes diagnosis date was used. These data were obtained from either the SCI‐Diabetes dataset or the Clinical Laboratory dataset, with the latter containing all biochemistry test results carried out within NHS Tayside and Fife over the study period. Time from pregnancy to diabetes diagnosis was calculated from the date of the first affected pregnancy for cases and the date of the last unaffected pregnancy for controls.

### 2.3 Diabetic complications

Diabetic complications were divided into microvascular complications—diabetic retinopathy, chronic kidney disease (CKD) and diabetic proteinuric kidney disease (PKD)—and macrovascular complications—major adverse cardiovascular event (MACE) and death from any cause.

The Scottish National Diabetes Retinal Screening database, the Scottish National Death Records dataset, the Scottish Morbidity Records for general/acute inpatient and day cases (SMR01) dataset^10^ and the Clinical Laboratory dataset were used to assess the risk of developing diabetic complications.

Diabetic retinopathy was defined as the earliest recorded date of any retinopathy (R1, mild background retinopathy to R4, proliferative retinopathy) using the Scottish Grading Programme for diabetic retinopathy,^11^ with data obtained from the Scottish National Diabetes Retinal Screening database. Chronic kidney disease (CKD) was defined based on the Kidney Disease: Improving Global Outcomes (KDIGO) creatinine‐based criteria, as described fully elsewhere,^12^ with data obtained from the Clinical Laboratory and SMR01 datasets. Proteinuric kidney disease (PKD) was defined as the earliest date of a urinary albumin: creatinine ratio (ACR) >30 mg/mmol or a urinary protein: creatinine ratio (PCR) >50 mg/mmol contained within the Clinical Laboratory dataset. MACE was defined as hospitalisation with stroke, hospitalisation with myocardial infarction and vascular death. The Scottish Morbidity Records for general/acute inpatient and day cases (SMR01)^10^ and Scottish National Death Records datasets were used to obtain this information. The ICD9 and ICD10 codes used to define the endpoints are described elsewhere.^13^ All‐cause death data were obtained from the Scottish National Death Records dataset.

### 2.4 Statistical methods

Univariate linear regression was used to determine the impact of a PE history on the aforementioned clinical variables—BMI, HDL cholesterol, total cholesterol, HbA1c and age—at the point of type 2 diabetes diagnosis, as well as the time between pregnancy and diabetes diagnosis. As all of these clinical parameters are physiologically interrelated, each independent clinical variable was adjusted for the other previously named clinical parameters using multivariable linear regression. For example, BMI was adjusted for HDL cholesterol, total cholesterol, HbA1c and age at type 2 diagnosis, whereas HDL cholesterol was adjusted for BMI, total cholesterol, HbA1c and age at type 2 diagnosis. This provided an independent assessment of the impact of a history of PE on the clinical status of a woman at the time of type 2 diabetes diagnosis.

Cox regression models were used to assess how a PE‐history influenced the risk of future microvascular complications from the time of type 2 diabetes diagnosis. These models were adjusted for age and HbA1c at the time of type 2 diabetes diagnosis. For macrovascular complications and death from any cause, Cox regression analysis was not carried out due to the inherent survival bias in our study data caused by the large time difference between the date of pregnancy and the date of diagnosis with diabetes, and subsequent registration within the SCI diabetes datasets. For this reason, we only consider the average age difference at the time of macrovascular complications and death between patients with and without a history of PE in at least one pregnancy.

## 3 RESULTS

### 3.1 Study numbers and demographics

**FIGURE 1:**
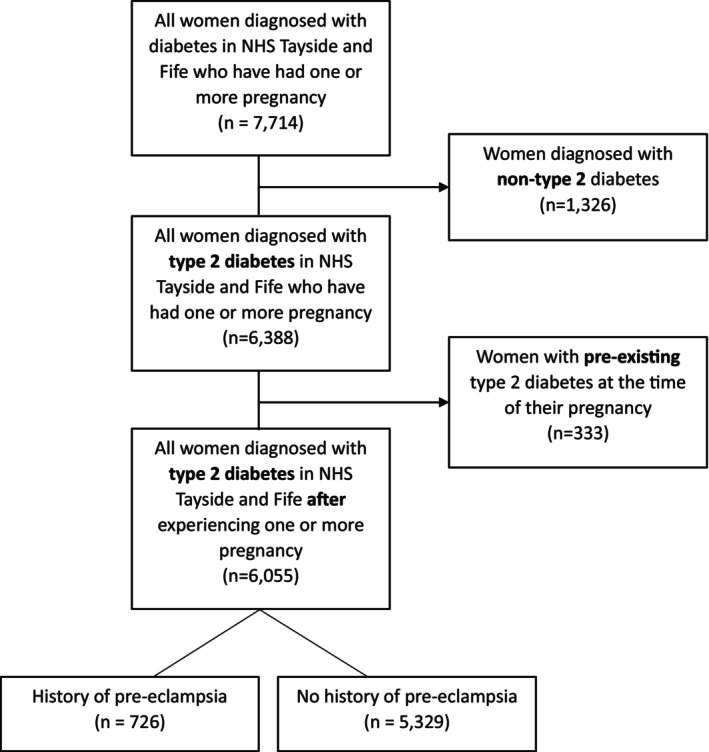
Study population flow chart—women from NHS Tayside and Fife with a diagnosis of type 2 diabetes and pregnancy records.

In total, 6338 women diagnosed with type 2 diabetes and registered in the SCI‐Diabetes data set also had information on an earlier pregnancy in the NHS Tayside and Fife Maternity records (SMR02) (Figure 1). In total, 333 women were excluded as they had type 2 diabetes prior to pregnancy, leaving 6055 women. Of these, 726 (12%) had a history of PE in at least one pregnancy and 5329 (88%) had no such history. Eleven (0.18%) had a history of eclampsia. All pregnancies >24 weeks—both single and multiple—were included. The median follow‐up time post‐partum was 35.6 years (IQR 25.1–42.7 years), and the median follow‐up time post type 2 diabetes diagnosis was 10.2 years (IQR 5.5–15.7 years).

### 3.2 Clinical status at diagnosis of type 2 diabetes

The clinical features of the women at the time of diagnosis are shown in Table 1. Compared to women with non‐PE pregnancies, those with a PE history had higher BMI (38.3 ± 7.8 kg/m^2^ vs. 35.7 ± 7.7 kg/m^2^, *p* < 0.001), higher systolic blood pressure (139 ± 14 mmHg vs. 135 ± 14 mmHg, *p* < 0.001) and lower HDL cholesterol (1.18 ± 0.25 mmol/L vs. 1.21 ± 0.30 mmol/L, *p* = 0.002) at the point of type 2 diabetes diagnosis. A history of PE had no association with total cholesterol or HbA1c levels at the time of type 2 diabetes diagnosis. Women with a pregnancy affected by PE were on average almost 4 years younger at diagnosis with type 2 diabetes, with a 1‐year shorter period between first affected pregnancy and diabetes diagnosis compared to controls (20.8 years vs. 22.0 years, *p* = 0.002).

**TABLE 1:** Clinical features of individuals at diagnosis of diabetes.

Variable		PE cases	[SD]	PE‐free controls	[SD]	Difference between cases and controls (univariate)	*p*‐Value (univariate)	Adjusted difference between cases and controls (multivariate)	*p*‐Value (multivariate)
		Mean		Mean					
Age at type 2 diabetes diagnosis	years	48.7	[9.2]	52.5	[10.5]	−3.82	<0.001	−3.26	<0.001
Time between pregnancy and diabetes diagnosis	years	20.8	[9.2]	22.0	[10.6]	−1.2	0.002	−1.07	0.011
Body mass index	kg/m^2^	38.3	[7.8]	35.7	[7.7]	2.61	<0.001	1.84	<0.001
Systolic blood pressure	mmHg	139	[13.8]	135	[13.6]	3.40	<0.001	2.02	<0.001
HDL cholesterol	mmol/L	1.18	[0.25]	1.21	[0.30]	−0.03	0.008	0.01	0.338
Total cholesterol	mmol/L	5.11	[0.94]	5.12	[0.96]	−0.016	0.697	−0.09	0.036
HbA1c	mmol/mol	56.9 (7.4%)	[13.7]	57.1 (7.4%)	[14.1]	−0.215	0.708	−0.38	0.545
CKD		26	(3.6%)	216	(4.1%)		0.707		

When adjusted for the other clinical variables—age, BMI, systolic blood pressure, HDL cholesterol, total cholesterol and HbA1c—in multivariable linear regression models as described above, the differences between women with a history of PE and women without a PE history at the time of type 2 diabetes diagnosis were only mildly attenuated (Table 2). A PE history remained independently associated with a younger age of diagnosis (3.26 years, *p* < 0.001) and shorter time between pregnancy and diabetes diagnosis (1.07 years, *p* = 0.011), as well as a higher BMI (2.61 kg/m^2^, *p* < 0.001) and systolic blood pressure (2 mmHg, *p* < 0.001) at the point of type 2 diagnosis.

**TABLE 2:** Hazard ratios for complications of type 2 diabetes in women with a history of pre‐eclampsia in pregnancy.

Outcome	Adjusted hazard ratio^a^	95% CI (lower)	95% CI (upper)	*p*‐Value
Chronic kidney disease (*n* = 618)	1.35	1.06	1.71	0.016
Proteinuric kidney disease (*n* = 32)	2.45	1.03	5.80	0.042
Diabetic retinopathy (*n* = 2036)	1.22	1.07	1.38	0.003
Any microvascular complication (*n* = 2305)	1.26	1.12	1.42	<0.001

^a^ Adjusted for age and HbA1c at the time of diagnosis of type 2 diabetes mellitus.

### 3.3 Microvascular complications

Following diagnosis with type 2 diabetes, a PE history was associated with a 35% increased risk of developing CKD (HR 1.35 95% CI 1.06–1.71, *p* = 0.016), 145% increased risk of developing PKD (HR 2.45 95% CI 1.03–5.81, *p* < 0.001) and a 22% greater risk of developing diabetic retinopathy (HR 1.22 95% CI 1.07–1.38, *p* = 0.003) when adjusted for HbA1c and age at diabetes diagnosis (Table 2) compared to women who had only PE‐free pregnancies. Incidence rates and absolute numbers are available in Table S3.

### 3.4 Macrovascular complications

The mean age at MACE occurrence was 4.9 years younger in women with a history of PE compared to women with no history of PE (55.7 years vs. 60.6 years, *p* < 0.0001), and 1.1 years younger when adjusted for the earlier age of diabetes diagnosis seen in women with a history of PE. Fatal cardiovascular events were the most common cause of death for both groups, causing 44% (35/80) and 47% (392/843) of deaths in the PE and non‐PE groups, respectively. For all‐cause death, women with a history of PE were 5.5 years younger compared to women with no PE history (60.1 years vs. 65.6 years, *p* < 0.0001), and 1.7 years younger when adjusted for the earlier age of diabetes diagnosis seen in women with a history of PE.

## 4 DISCUSSION

In a cohort of women diagnosed with type 2 diabetes after pregnancy, a history of PE was associated with a more severe cardiometabolic profile with higher systolic blood pressure, lower HDL cholesterol and a higher BMI at the time of diabetes diagnosis together with a 3.8‐year younger age of diagnosis of type 2 diabetes compared to women who did not experience PE. The difference in diagnosis age was only mildly attenuated (3.26 years) when adjusted for important risk factors—obesity, hypertension and an atherogenic lipid profile^14^—shared by both type 2 diabetes and PE, suggesting that PE is an independent risk factor for the development of type 2 diabetes.

As well as an earlier age of onset, women with a PE history were found to have a more severe clinical course of type 2 diabetes, with a greater risk for diabetic retinopathy, CKD and PKD. Interestingly, a PE history resulted in an overall 26% increased risk of developing microvascular complications post a type 2 diabetes diagnosis. There was no difference in mean HbA1c measurements between the two groups, suggesting that glycaemic control is not the main driver behind this observed increase. Further studies will be required to understand the molecular pathways underpinning the microvascular complications seen in women who experienced PE cases decades earlier.

There is a known association between PE and retinal involvement; PE frequently presents with visual changes ranging from blurred vision to blindness. Additionally, retinal imaging during pregnancy can be used to predict PE onset,^15^ with retinal changes similar to those observed in hypertensive retinopathy seen in severe PE.^16^ However, as with many of the other PE‐associated complications, it is increasingly recognised that any abnormal retinal vasculature changes^15^ leave the affected women at increased risk for retinal disease even decades post‐partum.^17^ Similarly, in mouse models, PE predisposed to a more rapid and severe retinopathy following a second cardiometabolic hit,^18^ such as type 2 diabetes. In our study, we showed that the risk of developing diabetic retinopathy in women with a PE history was 22% greater than in women with only PE‐free pregnancies.

Renal dysfunction during a PE episode is well documented; one of the defining features of PE is proteinuria secondary to renal endothelial cell dysfunction. Previous literature on the association between PE and future CKD is varied, with most studies showing an increased risk in those with a PEistory^3^, ^19^ but a minority finding no difference between affected and unaffected individuals.^20^ In our population with type 2 diabetes, we found that a PE history was associated with a 35% increased risk of developing CKD, and a marked 145% increased risk for PKD (HR 2.45). Due to the small numbers, the confidence intervals for this estimate are large. As shown in Table S3, the absolute risk difference is low; per 1000 women, there were only 5.5 additional cases of PKD in women with a history of PE (13.8 vs. 8.3). This indicates that the hallmark kidney dysfunction associated with PE, that is thought to resolve post‐partum, is either long‐lasting or leaves the affected women more susceptible to future damage. As the only study looking solely in a population of people with diabetes, it is difficult to draw direct comparisons with previously published literature; however, our findings align with the majority of previously published data that highlights the increased risks for CKD and diabetic PKD following a PE‐affected pregnancy.^21^

The increased CVD risk in women with a history of PE is similarly well‐established,^1^, ^22^, ^23^ and there is emerging evidence that even their offspring are at increased risk.^24^ Our study was unable to fully assess the association between a history of PE and CVD due to the inherent survival bias in our study design, where there was an average 20‐year lag between pregnancy and developing type 2 diabetes. As individuals with PE are at higher risk of CVD, they are more likely to have died from complications of these prior to developing type 2 diabetes and therefore not be included in our study. Despite this, we showed that women with a PE history had CVD events—fatal or non‐fatal—almost 5 years younger than women with no history of PE. Additionally, in our study, women with a PE history were dying more than 5 years earlier than controls from any cause. This is in line with studies that have shown that a history of PE‐affected pregnancy is associated with an increased long‐term mortality risk.^25^

Despite the recognised severity of PE—both at the time of pregnancy and decades post‐partum—the pathophysiology remains unclear. There is increasing recognition that endothelial dysfunction is not only a key feature at the time of pregnancy^26^ but may be long‐lasting, leading to a low‐grade chronic inflammatory state^27^ that could underlie many of the associated future complications. For example, both endothelial dysfunction and chronic inflammation are recognised risk factors for type‐2 diabetes.^28^ It is difficult, however, to ascertain whether this underlying endothelial dysfunction predates the PE episode, unmasked by the physiological stresses of pregnancy, or is a new feature in PE. We showed that the younger age of type 2 diabetes diagnosis in women with a PE history was independent of many of the shared metabolic risk factors; suggesting that the PE episode may inflict lasting damage that goes on to increase the risk of developing type 2 diabetes. However, this could also be explained by these women instead sharing risk factors for endothelial dysfunction that go on to predispose to PE, type 2 diabetes and the associated complications.

Our study highlights the essential requirement to keep women with a PE‐affected pregnancy under long‐term clinical review and, at present, the follow‐up for these women is generally limited. In the United Kingdom, the National Institute for Clinical Excellence (NICE) recommends that women with PE should be reviewed 6–8 weeks after birth to ensure that their hypertension and proteinuria have resolved, with no further follow‐up unless either persists.^29^ This guidance neglects the long‐term health impacts that an episode of PE is associated with, providing no recommendations for ongoing monitoring or additional screening. Annual blood pressure measurements and a regular assessment of cardiovascular risk factors such as serum lipids and blood glucose are recommended by the International Study for the Society of Hypertension in Pregnancy,^30^ but Australia and New Zealand alone offer this.^31^ To our knowledge, no country has yet implemented regular microvascular assessment post‐partum in women with a PE history. In addition, the initiation of more aggressive medical management of these high‐risk patients upon a type 2 diabetes diagnosis may help to mitigate against their increased risks for developing microvascular disease.

## 5 STRENGTHS AND LIMITATIONS

Strengths of our study include the large cohort size (~6000 patients) and long post‐partum follow‐up time (33 years mean overall). Additionally, extensive data linkage has allowed us to assess both the women's clinical status at the point of type 2 diabetes diagnosis and also a wide range of future complications. Other studies have looked at the association of a PE history with the development of CKD, retinopathy and CVD individually.^1^, ^17^, ^21^ However, to our knowledge, ours is the only study to provide a comprehensive overview of the increased risks that a woman diagnosed with type 2 diabetes faces if she has a history of PE in pregnancy.

The primary limitation of our study is that the patient cohort all have type 2 diabetes. Although PE is a risk factor for future type 2 diabetes, many women affected by PE will not go on to develop type 2 diabetes, and therefore our findings for the risk of future complications such as CKD and CVD cannot be applied to the general population. Additionally, with an average time from pregnancy to type 2 diabetes development of >20 years, our study excludes any patients who die prior to developing type 2 diabetes and therefore is subject to survivorship bias, particularly affecting macrovascular complication risk. Therefore, our study likely underestimates the associated risks, particularly of the more commonly fatal macrovascular complications such as CVD and CKD. Finally, it may be that women with a history of PE have more surveillance by the medical system, and hence get diagnosed earlier, although long‐term follow‐up of women post an episode of PE is not routine.

## 6 CONCLUSIONS

For a woman recently diagnosed with type 2 diabetes, a history of PE in pregnancy is highly relevant to her future care. Compared to equivalent women with no PE history, she is, on average, 3 years 3 months younger, with a 22% increased risk of developing diabetic retinopathy, a 35% increased risk of developing chronic kidney disease and in particular, a 145% increase in the risk of developing proteinuric kidney disease. Regular post‐partum follow‐up with assessment of vascular health may enable earlier identification and treatment of these risks and reduce the increased mortality rate currently observed. At the very least, all women must be asked about a history of PE at the time of their type 2 diabetes diagnosis, in order for clinicians to identify this high‐risk cohort and to help guide their future management.

## AUTHOR CONTRIBUTIONS

M. C. Pearson: study concept and design; acquisition of data; interpretation and analysis of data; drafting of the manuscript. H. Wang: acquisition of data; interpretation and analysis of data; review of the manuscript. C. E. Murdoch: study concept and design; interpretation and analysis of data; review of the manuscript. A. S. F. Doney: study concept and design; acquisition of data; interpretation and analysis of data; drafting of the manuscript.

## FUNDING INFORMATION

CEM research is supported by and is coordinator of European Union’s Horizon 2020 research and innovation program under the Marie Sklodowska‐Curie agreement No 765274, iPlacenta. AD is supported by IUK 10072163.

## CONFLICT OF INTEREST STATEMENT

The authors have no conflicts of interests to declare.

## Supporting information


Data S1.

## Data Availability

The data that support the findings of this study are not openly available for reasons of sensitivity but are available from the corresponding author upon reasonable request.
