# Supplementary material for: Clinical implications of a history of pre‐eclampsia in women with type two diabetes mellitus
Source: Diabet Med. 2025 Apr 16;42(6):e70046. doi: 10.1111/dme.70046 (PMC12080981; doi:10.1111/dme.70046)
Supplement: Supplementary file 1 — Data S1. [file DME-42-e70046-s001.docx]

*Supplementary Table 1:* Datasets

| **Data Set Name** | **Summary** | **Use** |
| --- | --- | --- |
| SMR02 (1) | Information on all maternity inpatient and day cases in Scotland | Formation of study population |
| Scottish Care Information for Diabetes Collaboration (SCI-Diabetes) (2) | Data pertaining to all individuals diagnosed with diabetes in Scotland  e.g. date of diagnosis, blood pressure readings, treatment modalities | Formation of study population  Clinical status at diabetes diagnosis |
| Biochemistry Data Set | All biochemistry test results for patients within NHS Tayside and Fife | Clinical status at diabetes diagnosis  Diabetic complications |
| SMR01 (3) | Information on all general / acute inpatient and day cases in Scotland | Diabetic complications |
| Scottish National Diabetes Retinal Screening Database | Data on all retinal imaging data carried out as part of the Scottish national diabetic retinal screening programme | Diabetic complications |
| National Records of Scotland (NRS)- Death | All registrations to the National Records of Scotland of deaths | Diabetic complications |

*Supplementary Table 2:* ICD Codes

| **ICD Code** | **Meaning** |
| --- | --- |
| ICD10 | |
| O11 | Pre-eclampsia superimposed on chronic hypertension |
| O14 | Pre-eclampsia |
| O15 | Eclampsia |
| ICD9 | |
| 6424 | Mild or unspecified pre-eclampsia |
| 6425 | Severe pre-eclampsia |
| 6426 | Eclampsia |
| 6427 | Pre-eclampsia or eclampsia superimposed on pre-existing hypertension |

*Supplementary Table 3:* Incidence rates for developing diabetic complications in women with and without a history of eclamptic disease in pregnancy

| **Outcome** | **Pre-eclampsia Cases** | | **Pre-eclampsia free Controls** | |
| --- | --- | --- | --- | --- |
|  | **Number** | **Incidence per 1000 people** | **Number** | **Incidence per 1000 people** |
| Chronic kidney disease | 84 | 116 | 591 | 111 |
| Proteinuric kidney disease | 10 | 13.8 | 44 | 8.30 |
| Diabetic retinopathy | 306 | 421 | 1883 | 353 |
| Non-fatal cardiovascular event | 77 | 106 | 575 | 108 |
| Fatal cardiovascular event | 35 | 48.2 | 392 | 73.6 |
| All-cause mortality | 80 | 110 | 843 | 158 |

**References**

1. Public Health Scotland. Maternity Inpatient and Day Case - Scottish Morbidity Record (SMR02) 2024 [Available from: <https://publichealthscotland.scot/services/national-data-catalogue/national-datasets/a-to-z-of-datasets/maternity-inpatient-and-day-case-scottish-morbidity-record-smr02/>.

2. SCI-DI. Scottish Care Information – Diabetes Collaboration (SCI-DC) 2015 [Available from: <https://www.sci-diabetes.scot.nhs.uk>.

3. Scotland PH. SMR01 general/acute inpatient and day case 2024 [Available from: <https://publichealthscotland.scot/services/national-data-catalogue/smr-data-manual/definitions-by-smr-record-section/smr01-generalacute-inpatient-and-day-case/>.
